# Neural circuits for binocular vision: Ocular dominance, interocular matching, and disparity selectivity

**DOI:** 10.3389/fncir.2023.1084027

**Published:** 2023-02-15

**Authors:** Jianhua Cang, Jieming Fu, Seiji Tanabe

**Affiliations:** ^1^Department of Biology, University of Virginia, Charlottesville, VA, United States; ^2^Department of Psychology, University of Virginia, Charlottesville, VA, United States; ^3^Neuroscience Graduate Program, University of Virginia, Charlottesville, VA, United States

**Keywords:** binocular vision, stereopsis, orientation selectivity, critical period, visual cortex, lateral geniculate nucleus, feedforward model

## Abstract

The brain creates a single visual percept of the world with inputs from two eyes. This means that downstream structures must integrate information from the two eyes coherently. Not only does the brain meet this challenge effortlessly, it also uses small differences between the two eyes’ inputs, i.e., binocular disparity, to construct depth information in a perceptual process called stereopsis. Recent studies have advanced our understanding of the neural circuits underlying stereoscopic vision and its development. Here, we review these advances in the context of three binocular properties that have been most commonly studied for visual cortical neurons: ocular dominance of response magnitude, interocular matching of orientation preference, and response selectivity for binocular disparity. By focusing mostly on mouse studies, as well as recent studies using ferrets and tree shrews, we highlight unresolved controversies and significant knowledge gaps regarding the neural circuits underlying binocular vision. We note that in most ocular dominance studies, only monocular stimulations are used, which could lead to a mischaracterization of binocularity. On the other hand, much remains unknown regarding the circuit basis of interocular matching and disparity selectivity and its development. We conclude by outlining opportunities for future studies on the neural circuits and functional development of binocular integration in the early visual system.

## Introduction

The three-dimensional visual world is projected as two-dimensional images onto the retina. The brain constructs depth information by comparing the small differences between the two flat retinal images, i.e., binocular disparity, in a perceptual process called stereopsis (Read, 2021). Stereoscopic vision is presumably mediated by neurons in the visual cortex that respond through the two eyes to the same (or similar) locations in visual space and selectively encode particular disparities (Cumming and Deangelis, 2001). These receptive field properties of cortical neurons are generated by precise anatomical projections leading to the primary visual cortex (V1) and exquisite neural circuits within the cortex, both of which are established by elaborate processes during embryonic and postnatal development.

**Figure 1:**
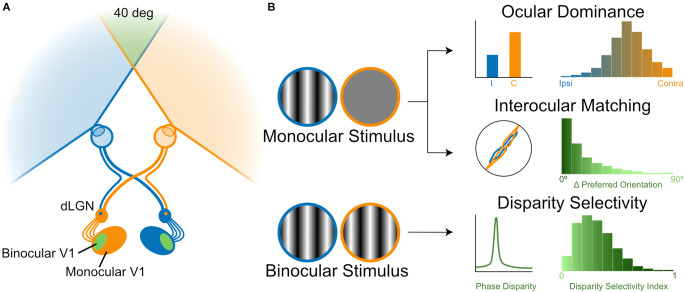
Binocular processing in the visual system. **(A)** A schematic diagram of the mouse visual system. The two eyes’ inputs are illustrated by a color code, which is followed in all figures. Retinal ganglion cell (RGC) axons either cross to the contralateral or stay on the ipsilateral hemisphere, leading to binocular interactions in downstream brain structures (dLGN: dorsal lateral geniculate nucleus; V1, primary visual cortex). **(B)** Three measures of binocular properties. Ocular dominance (OD) measures the relative response magnitude of individual neurons through the two eyes [top panel, a schematic representation of OD distribution of mouse V1 neurons, based on Gordon and Stryker (1996) and Mrsic-Flogel et al. (2007); whereas interocular matching quantifies the similarity of their tuning, such as orientation preference [middle panel, a schematic representation of the interocular difference between preferred orientations of mouse V1 neurons, based on Wang et al. (2010) and Wang et al. (2013). Both ocular dominance and interocular matching are studied by stimulating one eye at a time and then compared between the two eyes (left diagram). In contrast, the response selectivity for the binocular disparity is studied by stimulating the two eyes simultaneously with dichoptic gratings or random-dot stereograms [bottom panel, a schematic representation of disparity selectivity distribution of mouse V1 neurons in response to dichoptic gratings, based on Scholl et al. (2013) and Tanabe et al. (2022).

The first step of binocular vision is the crossing/uncrossing of retinal ganglion cell (RGC) axons at the optic chiasm. In primates including humans, RGCs in the temporal retina that view the contralateral half of the binocular visual field do not cross the chiasm and stay within the same hemisphere while projecting to the dorsal lateral geniculate nucleus (dLGN) of the thalamus, whereas nasal RGCs cross the chiasm and project to the dLGN of the other hemisphere (Figure 1A). As a result, each dLGN receives information about the contralateral visual field through both eyes, thus providing an anatomical basis for binocular interactions within the same hemisphere. The crossed and uncrossed axons from the two eyes remain segregated and form eye-specific layers in the dLGN. These layers each contain a retinotopic representation of the visual space, which is aligned with maps in the other layers. This arrangement eventually leads to binocular cortical neurons representing similar points in the visual space through the two eyes, after relay neurons of different dLGN layers converge onto V1 with topographically precise projections. The general organization of this binocular visual pathway is seen in all mammals that have been studied, even though the extent of binocular overlap, the ratio of crossed/uncrossed axons, and the number of layers in the dLGN differ across species (Petros et al., 2008). In mice, the main model organism discussed in this review, the binocular field is small (~40° in visual space, Figure 1A) because their eyes are positioned more laterally (Gordon and Stryker, 1996; Ibbotson and Jung, 2020). Only 3%–5% of RGCs in mice are uncrossed and project to the ipsilateral hemisphere (Petros et al., 2008). These RGCs originate from the ventral-temporal crescent of the retina, where they intermingle with contralateral-projecting RGCs (Pak et al., 2004), unlike the clearer decussation between crossed and uncrossed RGCs in more binocular animals (Lambot et al., 2005; Petros et al., 2008). Additionally, instead of the six-layer structure seen in primates, the mouse dLGN contains only a patch of ipsilateral RGC axons surrounded by contralateral terminals (Figure 1A). Despite these differences, the mouse has been a productive model in studying chiasm crossing and map formation/alignment during development. Studies have identified transcription factors, guidance cues, and transmembrane receptors, as well as their spatial and temporal patterns, in these developmental processes. Readers may refer to recent reviews on these topics (Petros et al., 2008; Cang and Feldheim, 2013), which will not be discussed further in this review.

Instead, we will focus our discussion on the physiology of binocular integration and its development in the visual thalamus and cortex. Given the segregation of eye-specific layers in the dLGN, it is thought that substantial binocular integration does not occur until the information reaches V1, even though binocular neurons have been reported in the dLGN of several species (more below in the section of “ocular dominance”). In carnivores and primates, the afferent terminals of the dLGN relay cells representing the two eyes continue to be segregated into the input-recipient layer of V1 (layer 4 in carnivores and layer 4c in primates), creating alternating bands of ocular dominance (OD) columns (Levay et al., 1975, 1978). As a result, neurons in the thalamus-recipient layer in these species are predominantly monocular, i.e., responding to only one eye. Neurons outside of layer 4 are binocular, receiving converging inputs from OD columns representing both eyes (Hubel and Wiesel, 1962). In mice, which do not have OD columns, the convergence of eye-specific afferents and consequently binocular neurons are seen at layer 4 (Gordon and Stryker, 1996; Figures 1A,B). In addition to binocular integration, another major transformation takes place when visual information reaches the cortex, where V1 neurons become selective for stimulus features such as orientation (Hubel and Wiesel, 1962). It has been shown in many species that binocular V1 neurons show similar orientation preference through the two eyes (Hubel and Wiesel, 1962; Nelson et al., 1977; Ferster, 1981; Bridge and Cumming, 2001; Wang et al., 2010; Figure 1B, middle panel), a feature presumably important for binocular integration. Finally, “disparity-tuned” neurons are first seen along the visual pathway in V1. These neurons respond preferentially to a range of binocular disparities that is geometrically equivalent to a particular depth (Figure 1B, bottom panel), and the subsequent spiking activity is thought to carry the signals required for stereoscopic depth perception (Barlow et al., 1967; Pettigrew et al., 1968; Cumming and Deangelis, 2001; Parker, 2007).

In this review, we will focus on these three properties of V1 neurons: ocular dominance of response magnitude, interocular matching of orientation preference, and response selectivity for binocular disparity (Figure 1B). These properties center around the two notable functions of the V1 circuitry, namely, orientation selectivity and binocular combination. By understanding their relationship, we can start to ask what circuit mechanisms exist for a computation that takes two raw forms of sensory signals and combines them into a signal that could later be used for perception and behavior. For other features of binocular vision, such as binocular suppression binocular rivalry, stereo correspondence, and comparison across species, readers are referred to recent reviews on these topics (Brascamp et al., 2015; Read, 2021; Maier et al., 2022).

## Ocular dominance and its plasticity

In their “first magnum opus,” Hubel and Wiesel (1962) characterized binocular responses in cat V1. The vast majority of the recorded cells were activated by stimuli to either eye, and their responses displayed different levels of dominance by one eye or the other. Hubel and Wiesel came up with a simple 7-group measure to describe the distribution of ocular dominance (OD). Groups 1 and 7 were neurons responding exclusively to contralateral and ipsilateral eyes, respectively. The intermediate groups (2–6) were deemed binocular neurons of different levels of OD, with group 4 showing the same or similar responses through the two eyes. Despite its qualitative nature, this classification scheme proved extremely useful in characterizing OD in many species and especially in quantifying changes after visual manipulations. Indeed, using this measure, Hubel and Wiesel demonstrated the existence of a critical period for binocular development in cats and monkeys (Wiesel and Hubel, 1963; Hubel et al., 1977). During the critical period, an imbalance of the two eyes’ input, such as that caused by monocular deprivation by lid suture in animal models or amblyopia (lazy eye) in children, leads to a shift of OD distribution away from the deprived eye and towards the non-deprived eye. This phenomenon is referred to as “OD plasticity,” which declines with age in postnatal development. Long-term deprivation that is not corrected before the closure of the critical period causes permanent deficits in spatial acuity through the affected eye and disrupted stereoscopic vision (Espinosa and Stryker, 2012). Later studies have extended the research of OD plasticity to many other species. These studies, especially those in mice and rats, have generated tremendous amounts of knowledge regarding OD plasticity and the regulation of its critical period. For example, one line of research aims to reveal deprivation-induced synaptic changes that lead to the initial depression of V1 responses to the deprived eye and the subsequent homeostatic potentiation to the open eye (Kaneko and Stryker, 2017). The other line aims to understand what controls the timing of the critical period. These studies have demonstrated that the maturation of specific inhibitory circuits in the visual cortex is important for both opening and closing the critical period, and they have also revealed a number of “molecular brakes” that restricts plasticity after the critical period and consequently approaches to “reopening” the window of plasticity in adulthood (Stryker and Lowel, 2018). It is beyond the scope of this article to provide a comprehensive review of molecular and synaptic mechanisms of OD plasticity and critical period regulation, on which there are plenty in recent publications (Espinosa and Stryker, 2012; Hensch and Quinlan, 2018; Hooks and Chen, 2020; Kasamatsu and Imamura, 2020; Xu et al., 2020a). We will therefore limit our discussion to two issues of OD that are the most relevant to binocular vision.

### Classification of binocularity in mouse V1

**Figure 2:**
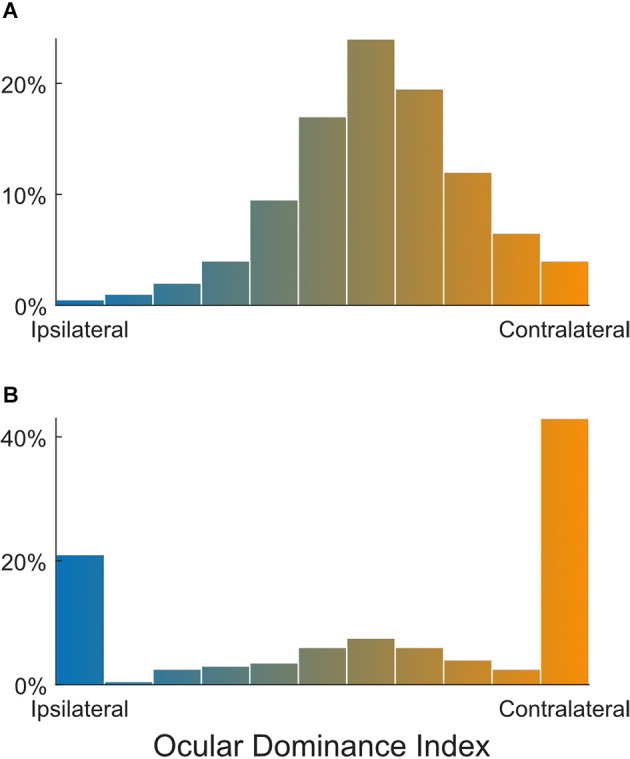
Different ocular dominance distributions reported in mouse V1. Whereas most studies have shown that the vast majority of neurons in the mouse V1 binocular zone are binocular **(A)**, some recent imaging studies reported a substantial population of monocular neurons **(B)**. Panel **(A)** is a schematic to illustrate that most V1 neurons have intermediate OD indices based on Gordon and Stryker (1996) and Mrsic-Flogel et al. (2007) and a number of other studies; whereas Panel **(B)** shows that large proportions of V1 neurons were reported to respond only to ipsilateral or contralateral stimulations, according to Salinas et al. (2017) and other recent 2-photon calcium imaging studies. The large monocular populations could be due to false negative statistical errors in how binocularity was determined (see Main Text for details).

Using the same method of classifying into seven groups, Gordon and Stryker (1996) characterized the OD of mouse V1 neurons. Within the binocular zone (~20°, either side of the vertical meridian), the response of mouse V1 neurons was heavily biased towards the contralateral eye, with many more neurons in groups 1–3 than in 5–7. Still, ~80% of the recorded neurons were groups 2–6, i.e., binocular. This OD distribution in normal unmanipulated mice was confirmed by many later studies, also using single unit recordings and the same OD measure or indices that are more quantitative by comparing the response magnitude through the two eyes (Figure 2A; e.g., Fagiolini and Hensch, 2000; Taha et al., 2002; Mcgee et al., 2005; Morishita et al., 2010). A similar OD distribution has also been seen in studies using 2-photon calcium imaging with synthetic calcium indicators (Mrsic-Flogel et al., 2007; Kameyama et al., 2010) or viral expression of calcium indicator GCaMP6 (La Chioma et al., 2020). However, a few recent 2-photon imaging studies using transgenic expression of GCaMP have reported very different distributions (Salinas et al., 2017; Huh et al., 2020; Jenks and Shepherd, 2020; Tan et al., 2020). They showed that, in mice after the critical period, fewer than 40% of responsive neurons in layer 2/3 of binocular V1 were “binocular,” whereas the others were all “monocular,” only responding to one eye (Figure 2B). The percentage of binocular neurons was even smaller for layer 4 neurons, only 7% in one study (Huh et al., 2020).

What could give rise to the discrepancy between these studies? It is certainly conceivable that these new studies were able to provide a more complete sampling within the binocular V1, whereas single unit recordings could have severely under-sampled the monocular population. It is also possible that this discrepancy was caused by the difference in sampling (e.g., mostly layer 2/3 neurons in imaging), as different layers may have different degrees of binocularity (Medini, 2011). However, these possibilities are unlikely given the huge difference between the reported numbers (~80% vs. <40%) and that other 2-photon imaging results were largely consistent with the single unit data. A more likely reason may be how ocularity was determined in these recent studies. These studies used monocular stimuli and then applied certain statistical criteria to determine responsiveness. Cells that passed the criteria in response to both (but separate) contralateral and ipsilateral stimulation were classified as “binocular.” Such a procedure possesses an inherent risk for type II error (false negative), which would lead to an underestimation of binocular neurons because it requires both monocular conditions to be met (in any studies that use such a procedure, not just limited to imaging). For example, for a population of 100 binocular neurons, a 20% false negative rate would lead to a conclusion of 80 responding to the contralateral eye, 80 ipsilateral, 64 of them to both (a 36% underestimation), and four non-responsive, assuming independent monocular responses. This issue may be further exacerbated by the use of GCaMP6 transgenic mice, which likely have a lower sensitivity of reporting spikes than viral transfections. Indeed, it was demonstrated that 2-photon calcium imaging often fails to detect spikes at low firing rates in GCaMP6 transgenic mice (Huang et al., 2021). As a result, a weak monocular response would likely be classified as non-responsive to that eye, thus leading to an underestimation of the binocular population.

Finally, it is important to emphasize that showing suprathreshold responses to only one eye does not necessarily make the cell “monocular.” Indeed, Hubel and Wiesel noted in their original article that “even if no response was obtained from the non-dominant eye, the two eyes were stimulated together in parallel to see if their effects were synergistic. With these methods, an influence was frequently observed from the non-dominant eye that might otherwise have been overlooked” (Hubel and Wiesel, 1962). These neurons would be included in groups 2 and 6, “in which the non-dominant eye, ineffective by itself, could influence the response to stimulation of the dominant eye.” Later studies confirmed the existence of such binocular “modifications,” which include both excitation and inhibition (Dougherty et al., 2019a). A recent study reported that in monkey V1, many of the “monocular” neurons, including those in the input layers, showed response modulation by the non-driven eye (Dougherty et al., 2019b). Such binocular modulations were mostly suppressive, suggesting the involvement of cortical inhibition in this process. Unfortunately, binocular stimulation is now rarely used in OD studies, which would lead to an underestimation of the binocular population in these studies (not just the recent imaging studies cited above). To correct this, a *bona fide* binocular stimulation system should be implemented, where stimuli to the two eyes can be independently controlled and simultaneously applied, such as dichoptic gratings (Ohzawa and Freeman, 1986) and random-dot stereograms (Poggio et al., 1985) used for studying binocular disparity selectivity, or those used in psychophysics experiments to study binocular rivalry (Brascamp et al., 2015). Such a stimulus system will provide a better estimation of binocular neurons in mouse V1, ideally with large-scale physiological recordings to avoid the sensitivity issue in 2-photon calcium imaging. More importantly, it will reveal the likely diverse binocular interactions in mouse V1 beyond ocular dominance and the simple “synergistic” effect seen in the original study of cat V1. Given the importance of mice in mechanistic studies of OD plasticity and other binocular properties, such a thorough characterization is urgently needed.

### Binocular interactions in dLGN

Another recent and surprising finding is the reports of OD plasticity in the mouse dLGN. This was unexpected because the LGN is often considered a monocular structure. However, binocular interactions have long been reported for dLGN neurons. In their review, Dougherty et al provided a detailed summary of such data based on extracellular recordings in anesthetized cats and monkeys (Dougherty et al., 2019a). Even though the percentage of dLGN neurons that respond to both monocular stimulations is small (“binocularly driven,” 3% in monkey; 2%–10% in cat), many more are “binocularly modulated” (i.e., the response to the driving eye is modulated by stimulation to the other eye). The modulation could be facilitation or suppression, with suppression more often than facilitation (~70% vs. 10% dLGN neurons in cats and 10%–30% vs. 5% in monkeys; Dougherty et al., 2019a).

Recent studies have indicated that the degree of binocular interactions may be even greater in the mouse LGN. In one study using multielectrode recordings in anesthetized mice (Howarth et al., 2014), while about two-thirds of the responsive neurons responded exclusively to contralateral stimulation of full field flashes, no cells exhibited purely ipsilateral responses. In other words, all ipsilaterally responsive cells in the mouse dLGN were also driven by contralateral stimulation. These binocularly driven neurons were mostly found at or around the patch of ipsilateral retinal projections in the dLGN, and their “binocularity” was unchanged upon cortical inactivation (Howarth et al., 2014). This result was largely confirmed by a later study of a smaller dataset (Sommeijer et al., 2017), which also used multielectrode recording and full-screen flashes and reported that “most single units in dLGN responding to the ipsilateral eye also responded to the contralateral eye.” In contrast, lower degrees of binocular integration were reported in studies that used 2-photon calcium imaging of dLGN axonal boutons in the superficial layers of V1. The percentage of binocularly driven boutons imaged in the V1 binocular zone was reported to be 14% (Jaepel et al., 2017) and 6% (Huh et al., 2020). In addition, purely ipsilaterally responsive boutons were observed in these studies, ranging from ~20% (Jaepel et al., 2017) to ~40% (Huh et al., 2020). The discrepancy between imaging and physiological recordings could again be due to false negatives in statistical analysis and lower sensitivity of calcium imaging. Indeed, Bauer et al. (2021) noted that the percentage of binocularly driven boutons ranged from 7% to 21%, “depending on the stringency of the statistical selection criteria used.”

Perhaps even more surprisingly, mouse dLGN neurons were shown to undergo OD plasticity, both during the critical period (Sommeijer et al., 2017; Huh et al., 2020) and in adult mice under environmentally enriched conditions (Jaepel et al., 2017). Following monocular deprivation, dLGN neurons, studied by single unit recording or 2-photon imaging of their axonal boutons, would reduce or lose their responses to the deprived contralateral eye and increase or gain responses to the non-deprived ipsilateral eye. The dLGN OD plasticity was largely resistant to cortical inactivation (Jaepel et al., 2017), but was compromised by reducing thalamic inhibition (Sommeijer et al., 2017).

What could give rise to binocular integration in the LGN? Using retrograde transsynaptic tracing to label RGCs that innervate individual dLGN neurons, Rompani et al. demonstrated direct retinal convergence in the mouse dLGN. They targeted “relay” or principal neurons in or near the ipsilateral patch to initiate the tracing. Out of the 25 dLGN neurons for which they identified presynaptic RGCs, 15 of them (60%) had input only from the contralateral eye, whereas the other 10 (40%) received inputs from both eyes. No purely ipsilaterally driven neurons were found, consistent with the *in vivo* recording data (Howarth et al., 2014). A later study developed an anterograde strategy to examine the strength of the converging retinogeniculate synapses, using optogenetics to activate retinal inputs in brain slices (Bauer et al., 2021). Most of the recorded dLGN neurons (~60%) received inputs from both eyes, but the vast majority of these binocular cells are dominated by one eye or the other. In fact, stimulation of the non-dominant eye’s input never triggered action potentials at resting membrane potential (Bauer et al., 2021). On the one hand, the limited “functional convergence” could explain the *in vivo* calcium imaging data showing large populations of purely monocular dLGN neurons despite the prominent anatomical binocular convergence (Bauer et al., 2021). On the other hand, the weak synapses from the non-dominant eye could provide a substrate for complex binocular interactions when both eyes are stimulated.

In addition to direct retinal convergence, inputs from other subcortical structures like the superior colliculus (SC) could also contribute to the binocularity in the dLGN. It was shown in anesthetized marmoset monkeys that the binocularly driven neurons were almost exclusively restricted to the koniocellular cells in the dLGN (Zeater et al., 2015). Koniocellular cells are located between the primary LGN layers and they uniquely receive input from the SC (Stepniewska et al., 1999). The SC receives both retinal and cortical inputs, and in primates, most SC neurons are binocularly driven (Moors and Vendrik, 1979). Diverse modes of binocular interactions are now also reported in the mouse SC (Russell et al., 2022). Whether these neurons project to the dLGN and contribute to the binocularity there is still unknown.

Together, despite some important discrepancies, these recent studies reveal a degree of binocular interactions in the mouse dLGN that was previously under-appreciated. What remains to be determined is how these interactions contribute to binocular vision and development. For example, it was shown that binocular neurons in the cat dLGN were not selective for binocular disparity (Xue et al., 1987), thus not encoding stereoscopic depth. Whether this is the case in the mouse dLGN, where binocular interactions seem more prominent, should be studied soon. Such information will be necessary for our understanding of the circuit basis of binocular integration in this widely used animal model.

## Interocular matching of orientation preference

As mentioned in the previous section, decades of studies have made OD plasticity and its critical period a classical model of experience-dependent neural development and amblyopia. However, it remained unclear what purpose critical period plasticity served during normal development. This is because OD plasticity is only induced by an imbalance of inputs from the two eyes, a condition that does not exist in normal visual system development. In fact, the degree of OD in V1, at least at the population level, does not change during the critical period unless the system is manipulated experimentally, such as by monocular deprivation (Sato and Stryker, 2008). What then does the heightened cortical plasticity, which is often revealed by OD plasticity, do when inputs from the two eyes are intact? Wang et al. (2010) hypothesized that it might allow the visual experience to drive the matching of orientation preference for V1 neurons through the two eyes. They tested this hypothesis in mice using single unit recoding and demonstrated that (1) the preferred orientations of individual V1 neurons are mismatched through the two eyes before the critical period, at postnatal day 20 (P20); (2) the interocular similarity of orientation preference improves and reaches adult levels by P30; and (3) alterations in visual experience during this period, but not in adulthood, disrupt the matching of orientation preference. These results, therefore, demonstrate that activity-dependent changes induced by normal visual experience serve to match eye-specific inputs in the cortex, thus revealing a functional purpose for the critical period in normal development. We referred to this process as “binocular matching” in previous publications, but it is worth noting that only monocular stimulations were used to determine orientation tuning for comparison between the two eyes. In other words, no binocular stimulation was delivered. Accordingly, “interocular matching” is a better term and will be used in this review.

Even with the mouse studies, it remained unclear whether experience-dependent interocular matching would also happen in other species, especially in those with a more advanced visual system. In primates and carnivore V1, cells with similar orientation preferences are organized into columns across cortical layers (namely orientation columns or orientation maps). This is different from mouse V1, where neurons are scattered and intermingled with others that are tuned to different orientations (i.e., a “salt-and-pepper” organization). Using intrinsic imaging to visualize such maps from either eye, early studies in cats actually showed that the matching of monocular maps could happen without normal visual experience (Godecke and Bonhoeffer, 1996; Crair et al., 1998). The difference between the results in mice and cats could reflect the species difference or could be due to methodological issues. A recent study revisited this topic in ferrets, another carnivore species with columnal organizations (Chang et al., 2020). They first used wide-field calcium imaging to examine the development of orientation maps and their interocular relationship. At eye-opening (~P30 in ferrets), stimulation of either eye revealed an adult-like orientation map. However, the two monocular maps were not matched at this age but did become matched about 1 week later. To examine whether this occurred at the cellular level, the authors used two-photon calcium imaging to determine the orientation preference of individual layer 2/3 neurons in ferret V1. Indeed, the number of mismatched neurons decreased during this period, just like in mice (Wang et al., 2010, 2013). Finally, delaying eye-opening by 1 week blocked interocular matching, and 1 week of visual experience after the deprivation did not completely rescue the matching deficit. In other words, normal visual experience during development is critical in generating a binocularly unified representation in ferret V1.

Together, the mouse and ferret studies indicate that interocular matching is likely a universal process that takes place during normal cortical development.

### Possible mechanisms for interocular matching

The circuit basis underlying interocular matching of orientation preference is still being actively investigated, but it is undoubtedly linked to the neural mechanisms of monocular orientation selectivity. According to the feedforward model proposed originally by Hubel and Wiesel (1962), orientation selectivity arises from specific arrangement of geniculate inputs, and the preferred orientation of individual cortical cells is determined by the layout of the elongated On and Off subregions in their receptive fields (RFs). This model has received experimental support in both cats (Reid and Alonso, 1995; Ferster et al., 1996) and mice (Li et al., 2013; Lien and Scanziani, 2013). Consistent with this model, it was shown in mice that simple cells match their orientation preference before complex cells (Wang et al., 2013), with the two classes of cells representing consecutive stages of cortical processing in the feedforward hierarchy (Ferster and Miller, 2000; Priebe and Ferster, 2012). In addition, the two monocular RFs show a correspondence between their structures, with a significant overlap between the same sign subregions (On–On and Off–Off). The RF subregion correspondence and consequently matching of RF orientation were disrupted (but not completely abolished) in mice deprived of visual experience during development (Sarnaik et al., 2014), indicating both experience-dependent and -independent processes. Finally, using optogenetic silencing and intracellular whole-cell recording, Gu and Cang (2016) isolated thalamic and cortical excitatory inputs to individual layer 4 neurons and studied their interocular matching. In adult mice, the thalamic and cortical inputs serving the same eyes are tuned to similar orientations and are both matched, consistent with the feedforward model for both monocular pathways. In young mice (P15–21), when the recorded neurons were still completely mismatched in their orientation tuning, their thalamic inputs were already slightly matched. In other words, the interocular matching of thalamic inputs initiates before that of intracortical circuits. Additionally, the intracortical circuits serving the contralateral eye appear to mature before those for the ipsilateral eye (Gu and Cang, 2016). Together, these results indicate that both thalamocortical and intracortical circuits undergo experience-dependent changes to ensure interocular matching and that these changes follow particular developmental profiles in a feedforward manner.

**Figure 3:**
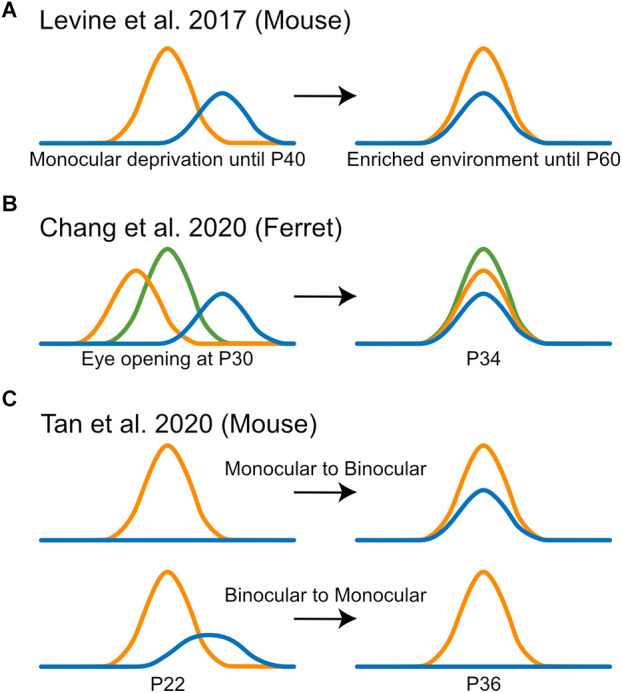
Observations from recent imaging studies of interocular matching of orientation preference. **(A)** Levine et al. (2017) imaged the recovery of interocular matching in mice after visual deprivation and found that for neurons that were clearly dominated by one eye, the input representing the weaker eye (blue) tended to change its orientation preference to align with that of the dominant eye (orange), which itself remained relatively constant. **(B)** Chang et al. (2020) imaged the interocular matching in ferrets after eye opening and found that the binocular map acted as a template for matching and individual neurons shifted their monocular orientation preferences (blue and orange) to reduce interocular mismatch, without losing their orientation selectivity or binocular responses (green). **(C)** Tan et al. (2020) found that there was a considerable turnover between “binocular” and “monocular” populations in mouse V1 from P22 to P36, where highly tuned monocular neurons tended to gain matched responses from the other eye (top) and poorly tuned binocular neurons tended to lose responsiveness to one eye (bottom).

How do V1 neurons change their monocular orientation preferences to match between the two eyes? Would the tuning through one eye change to match that through the other eye, or do they meet somewhere in the middle? At the heart of these questions is the need to reveal the logic or rules that govern the outcome of interocular matching. To address this, several recent studies have used 2-photon calcium imaging, which allows chronic tracking of the same cells over weeks or even months. First, Levine et al. (2017) imaged mice after visual deprivation that spanned the entire critical period (Figure 3A). As implied by the term of “critical period,” the disrupted matching did not fully recover without intervention. However, 3 weeks of environmental enrichment was sufficient to rescue interocular matching to the level seen in un-manipulated mice (Levine et al., 2017). This made it technically easier to follow matching than in younger mice during normal development. It was found that for layer 2/3 neurons that were clearly dominated by one eye, the input representing the weaker eye tended to change its orientation preference to align with that of the dominant eye, which itself remained relatively constant (Levine et al., 2017). These results suggest that Hebbian mechanisms may mediate matching recovery, where the dominant input instructs the weaker input to adopt its tuning properties. Indeed, a computational model that is based on a spike-timing-dependent plasticity (STDP) rule was able to recapitulate these experimental findings (Xu et al., 2020b). Whether this process also happens during normal development, and what predicts the matching outcome for cells with a balanced OD, remain unknown.

The study that demonstrated interocular matching in ferrets also followed the matching process using chronic imaging (Chang et al., 2020). At eye-opening, binocular stimulation generated an orientation map that was different from the monocular maps. The binocular map was significantly more stable during the week after vision onset when the two monocular maps became more similar to the binocular map. In other words, the binocular map acted as a template during matching. At the cellular level, individual layer 2/3 neurons shifted their monocular orientation preferences to reduce mismatch, without losing their orientation selectivity or binocular responses (Figure 3B). Together, these results suggest that in ferret V1, it is the neuron’s binocular responses that determine the matching outcome, again consistent with a correlation-based plasticity rule. Future studies are needed to reveal what determines the binocular responses at eye-opening, which would have important implications for understanding the circuit basis underlying interocular matching (Skyberg et al., 2020).

Finally, a series of articles by Tan et al. (2020, 2021, 2022) used 2-photon chronic calcium imaging to follow mouse V1 development from eye opening to after the critical period. These studies imaged transgenic mice expressing GCaMP6 in layer 2/3 or layer 4 neurons and used statistical criteria to classify binocular and monocular neurons. As mentioned earlier (see the Section of “*Classification of binocularity in mouse V1*”), much fewer binocular neurons were reported in these articles than in previous studies, where the percentage grew gradually from 3% to 18% during the developmental time window P14 to P36 (here, the percentages were out of the whole population instead of the responsive ones). By analyzing these selected populations, they showed that “binocular” neurons were largely mismatched at P14. Surprisingly, near adult levels of matching were achieved by P18, with only minor improvements through P36 (Tan et al., 2022). This was quite a bit earlier than reported in previous studies, which showed a largely random matching by ~P20 using single unit and intracellular whole cell recordings (Wang et al., 2010, 2013; Gu and Cang, 2016). This discrepancy is likely due to the false negative statistical errors in classifying binocular neurons as described above, which would lead to an under-estimation of the true binocular population, especially those weakly responsive and mismatched neurons. The imaging experiments further showed that from P14 to P18, some unresponsive and monocular neurons gained responses to become binocular, in a process that required normal visual experience (Tan et al., 2021). Finally, from P22 to P36, they reported a considerable turnover between the “binocular” and “monocular” populations (Figure 3C), which appeared to depend on the selectivity of individual neurons (Tan et al., 2020). However, these observations depend strongly on the binary classification into “binocular” vs. “monocular.” The developmental process needs to be characterized in a more nuanced and quantifiable way in order to reconcile the discrepancies across multiple studies.

In summary, interocular matching is now an established paradigm for studying experience-dependent neural development. As mentioned, OD plasticity is a manipulation-induced plasticity, where cells lose responses to the deprived eye as an adaptation to pathological conditions. In contrast, interocular matching is a normal vision-induced plasticity and leads to a beneficial outcome. This is consistent with the notion that experience-dependent processes are especially important for wiring up circuits that integrate different streams of information, such as in multimodal sensory integration and language development, where setting up the underlying neural circuits entirely by genetic programs is impossible (Cang and Feldheim, 2013). Consequently, deficits in critical period plasticity could lead to problems in language, cognitive, and social development, as seen in many neural developmental disorders (LeBlanc and Fagiolini, 2011). Indeed, the timing of critical period plasticity and, more importantly, the interocular matching of orientation preference are disrupted in MeCP2-null mice, a mouse model of Rett syndrome (Krishnan et al., 2015). Interocular matching will likely serve as a more functionally relevant model in the study of critical period plasticity in normal and diseased conditions, and much remains to be investigated for its underlying circuit mechanisms.

## Binocular disparity selectivity

**Figure 4:**
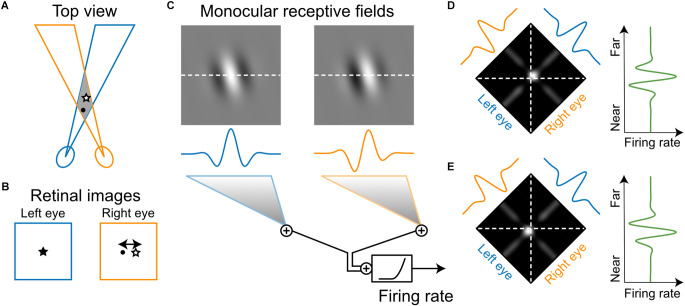
Model of how a neuron’s monocular receptive fields determine its selectivity to stereoscopic depth. **(A)** Many neurons in V1 have receptive fields in both eyes. Under normal binocular alignment, there is a region of space where the left- and right-eye receptive fields overlap. **(B)** A given object within the overlapped space does not necessarily project to the same position on both retinas (i.e., binocular disparity). An object close to the subject (circle) has a different binocular disparity than an object farther away (star). **(C)** Monocular receptive fields of a simple-cell-like neuron illustrated as patches of a 2D Gabor function, with oriented On (bright) and Off (dark) subregions (top). Cross sections of the receptive fields along the horizontal axis are illustrated below (middle). Various response properties of V1 neurons to binocular stimulation have been explained by a simple integration model: a linear summation of the monocular signals followed by an output nonlinearity (bottom). **(D)** The model makes specific predictions of how the neuron would respond to a small stimulus presented at various positions in the overlapping region of space (i.e., binocular interaction receptive field). When the stimulus is bright, the model predicts a small “hot spot” in space where the neuron would respond very strongly (left). That spot is where the on subregions in the left- and right-eye receptive fields overlap. When the stimulus is extended in space as in a random-dot pattern and is given a certain disparity, the predicted response will be a weighted summation of the binocular interaction receptive field along the frontoparallel axis. A gradual shift in the stimulus disparity will result in a continuous change in the neuron’s response (right). **(E)** Another neuron might have a different spatial offset between the two monocular receptive fields. The predicted hot spot for this neuron shifts to the near side of the frontoparallel plane (left), with the predicted disparity tuning having a peak on the near side (right).

In binocular animals, the images projected on the two retinas are slightly different due to the offset in the two eyes’ vantage points. The exact difference between the retinal images, namely binocular disparity, is determined by the geometry of the depth structures of the environment (Figures 4A,B). Binocular disparity, therefore, provides a powerful cue, which the visual system can use to represent and extract the depth of the three-dimensional world (Cumming and Deangelis, 2001). Barlow et al. (1967) were the first to discover neurons that were tuned to particular disparities in cat V1. By hand-mapping monocular response fields and analyzing binocular interactions, they reported that binocular neurons responded most strongly when the stimulus was located “correctly” in the visual fields of both eyes, i.e., of the appropriate disparity for that neuron. Such a binocular stimulus was more effective than a monocular stimulus, and “much more effective” than a binocular stimulus that was only positioned correctly in one eye. In other words, “the response to the correctly located image in one eye is vetoed if the image is incorrectly located in the other eye.” These findings were confirmed shortly after by Pettigrew et al. (1968), who used a variable prism to control the disparity of the stimulus for the two eyes. They found that most cat V1 neurons showed summation or facilitation of the monocular responses under optimal binocular disparity, but binocular occlusion when the prism setting was changed from the optimal value (Pettigrew et al., 1968).

Following these groundbreaking discoveries, a major goal in the field has been to study the circuit mechanisms underlying how monocular inputs are transformed into disparity-selective signals. This is not only essential for understanding the neural basis of stereoscopic vision, but also an excellent model for revealing general principles of cortical computation, where the monocular inputs are clearly defined, and the binocular output is an emergent selectivity that is important for the animal’s perception. Below we summarize the current understanding of disparity selectivity and review recent advances that may move the field forward.

### Disparity energy model

Ohzawa et al. (1990, 1996, 1997) performed a series of elegant studies in cats to systematically investigate the relationship between V1 neurons’ monocular RFs and their binocular disparity selectivity (Deangelis et al., 1991). The relationship was sufficiently explained by a simple, purely feedforward model, which is referred to as the “disparity energy” model. The word “energy” in the name derives from the model’s mathematical analogy with spatiotemporal energy models for motion perception (Adelson and Bergen, 1985). The front-end of the model is comprised of monocular RFs that correspond to the processing upstream of the neuron. Each of the monocular inputs, which contains segregated On and Off subregions (Figure 4C), is essentially the same feedforward organization proposed by Hubel and Wiesel (1962) to explain the emergence of orientation selectivity in V1 simple cells. Importantly, the two monocular RFs can have different degrees of spatial difference (phase difference and/or position offset) for individual neurons. The monocular signals are then combined, which is followed by an output nonlinearity (Figure 4C). Together, the spatial offset between the monocular RFs and the output nonlinearity makes the neuron sensitive to binocular interactions. Only specific disparities between left-eye and right-eye stimulation that are well matched with the RF offset would cause it to discharge, thereby generating disparity selectivity in V1 simple cells (Figures 4D,E). Subsequently, a number of such simple cells with different RF structures but similar disparity selectivity would converge to produce disparity-selective complex cells that are invariant to stimulus position or On/Off polarity (Ohzawa et al., 1990).

The disparity-energy model makes specific predictions of RF interactions, as well as disparity tuning characteristics to a variety of visual stimuli. A number of these predictions have been borne out with experimental data using monkeys (Cumming and Parker, 1997; Prince et al., 2002; Tsao et al., 2003). In addition, as mentioned earlier, the original feedforward model is supported by many studies in both cats and mice, providing a monocular RF basis for the disparity energy model. However, there has been very little data directly testing the predicted connectivity, presumably due to the limited availability of techniques for circuit dissection in the existing model organisms for binocular disparity (i.e., monkeys and cats). In the only study that examined connectivity patterns of disparity-selective neurons (Menz and Freeman, 2004), the authors performed paired recordings in cat V1 and found a variety of connectivity patterns—some were consistent while others were inconsistent with the model. However, it is difficult to examine connectivity based on cross correlation and the sample size was rather small, making it difficult to draw any definitive conclusion. Rather, this study underscores how little we know about the precise circuits for the generation of disparity selectivity and intracortical connections between disparity-selective simple and complex cells. Related, the model predicts that the essential connections for binocular combination are excitatory, whereas synaptic inhibition plays little or no role in this process. Whether this is true, and more generally inhibition’s role in disparity computation, remains unknown.

### Recent binocular disparity studies

Recent studies of binocular disparity have started to use mice, taking advantage of the available genetic, viral, and imaging techniques in this species. Disparity selectivity has been studied in mouse V1, first using single unit recording (Scholl et al., 2013) and later 2-photon calcium imaging (Scholl et al., 2015; Samonds et al., 2019; La Chioma et al., 2019, 2020). The early experiments used dichoptic gratings with a phase difference between the two eyes, which is useful to probe disparity tuning but does not have a direct relationship with depth, because the repeating pattern of a grating makes the binocular correspondence ambiguous (Fleet et al., 1996). The later imaging experiments also used random dot stereograms, which can reveal a neuron’s true selectivity for stereoscopic depth (Samonds et al., 2019; La Chioma et al., 2019, 2020). These studies reported substantial populations of disparity-selective neurons in mouse V1, as well as in higher visual areas. Interesting differences were observed between V1 and higher areas in terms of the tuned disparities (far vs. near; La Chioma et al., 2019) and shape (width and symmetry) of individual tuning curves (La Chioma et al., 2020). Some spatial clustering (~10 μm), but not large-scale organization, was seen for neurons tuned to similar disparities, which also showed higher noise correlations than differently tuned neurons (La Chioma et al., 2020). The observed range of tuned disparities is consistent with the behavioral performance of both head-fixed mice during a depth-discrimination task (Samonds et al., 2019) and freely moving mice in a pole descent cliff task (Boone et al., 2021). It should be noted that mice did not make vergence eye movements in response to different disparities (Samonds et al., 2019), consistent with the fact that mice do not have a fovea. Despite this difference with primates, mice could still be a useful model in studying the neural circuits underlying disparity computation because of the many available techniques. For example, it has been shown that Parvalbumin-expressing (PV+) inhibitory interneurons in mouse V1 had weaker disparity selectivity than PV- cells (Scholl et al., 2015). This is reminiscent of the observation that GABAergic neurons, especially fast-spiking ones, are more binocular than excitatory neurons (Yazaki-Sugiyama et al., 2009; Kameyama et al., 2010). PV+ neurons appear to be summing the output signals of excitatory neurons in their vicinity and could inhibit them in return. These results are suggestive of a negative feedback circuit for disparity computation, which is a substantial departure from the predictions made by the disparity-energy model.

These mouse studies indicate that with an additional model organism that facilitates more circuit-based analyses, new questions and understandings will arise regarding the actual circuit mechanisms of stereoscopic vision. Along the same line, we have recently studied binocular disparity selectivity in another animal model, the tree shrew (Tanabe et al., 2022). Tree shrews are a close relative of primates, and accordingly, their visual system shares various features with primate visual systems, such as six eye-specific layers in the dLGN (Conley et al., 1984) and a well-developed columnar architecture in the cortex (Bosking et al., 2002). Being diurnal and living in arboreal environments, tree shrews appear to require a sophisticated depth computation. Indeed, we found that tree shrew V1 neurons display highly selective responses to random dot stereograms, at a degree much higher than mice. Interestingly, both mouse and tree shrew V1 neurons show similarly strong disparity tuning to dichoptic gratings. The stimulus-dependent dissociation of disparity tuning between gratings and random dots is inconsistent with pure feedforward disparity energy model (Figure 4C). Instead, a simple network model, combining both feedforward and recurrent connections, can reproduce the essence of our observations in the two species (Tanabe et al., 2022). This model suggests that orientation-specific connectivity of excitation and inhibition could produce tree shrew-like tuning, whereas nonspecific connectivity could produce mouse-like tuning, thus raising an exciting hypothesis that orientation columns may play a crucial role in generating disparity selectivity. The validity of the model and more generally, the role of cortical circuits in disparity computation will need to be tested in future experiments. Excitingly, modern neuroscience techniques such as cell-type specific imaging and manipulation are being developed and applied in tree shrews with better success than in primates (Lee et al., 2016; Sedigh-Sarvestani et al., 2021; Schumacher et al., 2022). Tree shrews will therefore be a particular useful animal model in such studies (Savier et al., 2021).

### Disparity selectivity vs. other binocular properties

Although the three measures of binocular vision have been extensively studied in isolation, only a handful of studies have examined their relationship. In fact, the relationship between disparity selectivity and interocular matching has not been examined in any studies, and consequently, whether a high degree of matching is needed for disparity selectivity is unknown. On the other hand, Read and Cumming quantified OD using monocular stimuli and disparity selectivity using random dot stereogram in awake monkey V1 (Read and Cumming, 2004). They found no correlation between individual neurons’ OD and the strength or shape of their disparity tuning curves. In fact, even “monocular” neurons showed similar levels of disparity selectivity compared to the binocular ones. This is inconsistent with the disparity-energy model, which predicts that more strongly tuned neurons should have a more balanced OD. The lack of correlation between OD and disparity selectivity has been confirmed in cats and mice using dichoptic gratings (Kara and Boyd, 2009; Scholl et al., 2013; La Chioma et al., 2020). However, at the level of map organization, an interesting spatial relationship was seen between OD and disparity selectivity in cat area 18 (Kara and Boyd, 2009). The disparity selectivity is organized in a map where the preferred disparity of individual neurons (i.e., interocular phase difference) shows a smooth gradient across the cortex, and the disparity map seems to be orthogonal to the OD map (Kara and Boyd, 2009). The implication of this spatial organization for binocular integration remains unknown.

## Conclusions and perspectives

Despite the great progress that has been made, our understanding of the circuit and developmental mechanisms underlying binocular vision is still rather limited. We have pointed out several knowledge gaps throughout this review. Below we revisit some of these issues to emphasize their importance, as well as pointing out opportunities for future studies.

First, *binocular stimulation* is needed to study binocular vision. Binocular stimulation may not be necessary for most OD plasticity studies, but it is absolutely needed for understanding the diverse and complex binocular interactions along the visual pathway. Accordingly, classifying neurons into “monocular” and “binocular” is a complicated matter, where the type of visual stimuli, measurement sensitivity, and statistical criteria should be carefully considered. With these in mind, a thorough characterization of binocular interactions in mice, from dLGN to V1 to higher visual areas, will be needed to address many of the conflicting results in the literature.

Second, the *function of inhibition* has not been studied much for interocular matching or disparity selectivity. This is in drastic contrast to OD plasticity and its critical period, where the role of subtypes of cortical inhibitory neurons has been extensively documented. As mentioned, mouse PV+ interneurons show weaker disparity selectivity than PV- neurons (Scholl et al., 2015), and recurrent connections, including inhibitory circuits, are needed to explain the observed difference in disparity selectivity between mice and tree shrews (Tanabe et al., 2022). The exact contribution of inhibitory circuits to disparity computation remains to be revealed. For interocular matching, it was shown that suppressing somatostatin (SST+) interneurons during the critical period was able to block matching (Yaeger et al., 2019). However, it is unclear whether SST+ neurons were directly involved in the matching process, or if this outcome was due to their effect on cortical plasticity. The available “circuit-busting” techniques, such as cell type specific imaging and manipulations, will facilitate future studies in this exciting area.

Third, different *cortical layers* represent different stages of computation in visual processing. Studies in cats revealed that OD shifts induced by monocular deprivation appear in extragranular layers before in layer 4, suggesting that thalamocortical plasticity may be guided by earlier changes at higher stages (Trachtenberg et al., 2000). In contrast, interocular matching follows a feedforward sequence, with thalamocortical inputs matching before intracortical circuits (Gu and Cang, 2016) and simple cells before complex cells (Wang et al., 2013). However, layer-specific changes in interocular matching have not been reported. Similarly, not much is known about layer-specific computation of disparity selectivity. Future studies are needed to investigate interocular matching and disparity selectivity across all layers in order to understand their circuit mechanisms. Such studies will benefit from large-scale physiological recording as most imaging techniques are still largely restricted to superficial layers. Furthermore, callosal projections between the two hemispheres, which are layer specific in their targeting, have been shown to boost responses to the ipsilateral eye (Cerri et al., 2010; Dehmel and Löwel, 2014). However, their potential roles in interocular matching or disparity selectivity have not been studied much, though a lesion study suggested a limited contribution of callosal projections to binocular depth perception (Timney et al., 1985).

In addition, the *normal development of disparity selectivity* is almost completely unknown. Observations in human patients and deprivation studies in animals have clearly demonstrated that the development of stereoscopic depth perception takes place in a critical period in an experience-dependent manner. Upon monocular deprivation (amblyopia) or ocular misalignment (strabismus) in the critical period, almost all V1 neurons in primates and cats become completely monocular, thus lacking binocular integration. In mouse V1, monocular deprivation during the critical period weakens binocular disparity selectivity (Scholl et al., 2017). For normal development, one study reported the time course of V1 neuron disparity selectivity in developing monkeys and observed adult-like selectivity at postnatal day 6 (Chino et al., 1997). Similarly, another study described that disparity selectivity in cat V1 mostly developed before 3 weeks of age (Freeman and Ohzawa, 1992). The underlying circuit changes during development have not been studied, mirroring the lack of understanding of disparity computation in adult animals.

Finally, *diverse model organisms* are needed to advance the understanding of binocular vision and its development. Cats and monkeys were the models of choice for many years, providing many classical experiments and groundbreaking discoveries. Mice are, and will likely continue to be, a useful model in studying binocular vision, due to their powerful technical advantages. However, the mouse visual system differs from that of primates across an array of features, including the lack of a fovea, much lower spatial acuity, and different organizational features in the dLGN and V1. Consequently, binocular vision in mice, especially for disparity computation, may be implemented differently. Additional animal models, such as ferrets, tree shrews, and others, will be extremely useful due to their similarities to primates. Perhaps more importantly, comparisons between all these animal models will likely give rise to a much deeper understanding of this important neural computation.

## Author contributions

JC wrote the first draft of the manuscript with extensive discussion with JF and ST. JF and ST made the figures with inputs from JC. All authors edited and finalized manuscript. All authors contributed to the article and approved the submitted version.
